# Intersegmental Coordination in the Kinematics of Prehension Movements of Macaques

**DOI:** 10.1371/journal.pone.0132937

**Published:** 2015-07-15

**Authors:** Luisa Sartori, Andrea Camperio-Ciani, Maria Bulgheroni, Umberto Castiello

**Affiliations:** 1 Dipartimento di Psicologia Generale, University of Padova, Padova, Italy; 2 Cognitive Neuroscience Center, University of Padova, Padova, Italy; 3 Dipartimento FISSPA, University of Padova, Padova, Italy; 4 Ab.Acus., Milano, Italy; 5 Centro Linceo Interdisciplinare Beniamino Segre, Accademia dei Lincei, Roma, Italy; University of Chicago, UNITED STATES

## Abstract

The most popular model to explain how prehensile movements are organized assumes that they comprise two “components”, the reaching component encoding information regarding the object’s spatial location and the grasping component encoding information on the object’s intrinsic properties such as size and shape. Comparative kinematic studies on grasping behavior in the humans and in macaques have been carried out to investigate the similarities and differences existing across the two species. Although these studies seem to favor the hypothesis that macaques and humans share a number of kinematic features it remains unclear how the reaching and grasping components are coordinated during prehension movements in free-ranging macaque monkeys. Twelve hours of video footage was filmed of the monkeys as they snatched food items from one another (i.e., snatching) or collect them in the absence of competitors (i.e., unconstrained). The video samples were analyzed frame-by-frame using digitization techniques developed to perform two-dimensional post-hoc kinematic analyses of the two types of actions. The results indicate that only for the snatching condition when the reaching variability increased there was an increase in the amplitude of maximum grip aperture. Besides, the start of a break-point along the deceleration phase of the velocity profile correlated with the time at which maximum grip aperture occurred. These findings suggest that macaques can spatially and temporally couple the reaching and the grasping components when there is pressure to act quickly. They offer a substantial contribution to the debate about the nature of how prehensile actions are programmed.

## Introduction

A large number of detailed studies of the kinematics of human movement have provided evidence for the relative independence of reaching and grasping components in human prehension [1–7]. According to the majority of those studies, the reaching component encodes information regarding the object’s spatial location from the visual world and those muscles relevant to carrying the hand towards it are activated. The grasping component, instead, encodes information on the object’s intrinsic properties such as size and shape and the distal muscles involved in grasping the object of interest are activated [1–7].

Although the two components can be considered as distinct, they seem to be coupled functionally. Whereas the reaching component is primarily concerned with the function of coding object’s spatial location, object’s size could also modify this component [3, 4]. Similarly, whereas the grasping component serves primarily the function of gripping the target object, object’s spatial location could also modify this component [3, 4]. The standard parameters utilized for characterizing the reaching and the grasping components are essentially the time and amplitude of peak velocity and the time and amplitude of maximum grip aperture [1–7]. For instance, increasing the distance from the target brings to corresponding increases in reach peak velocity and the time to achieve maximum grip aperture is delayed [3, 4]. Similarly, as object size increases there is a corresponding increase in the reach peak velocity and the time of maximum grip aperture is reached at a later time [3, 4].

In view of their evolutionary implications, some investigators have performed a detailed kinematic analysis of the grasping behaviors of the macaque monkey, an animal model that is often used to study the neuronal mechanisms underlying the control of reach-to-grasp movements in humans (for review, see [8]). Indeed the evolution of the hand has been paralleled by changes in the primate central nervous system, in which the proportion of motor and sensory cortical areas devoted to the hand has massively expanded. The primate brain is endowed with a number of unique features, which testify to the high degree of specialization of hand movements in these species. First, the hand muscles’ motorneurons are monosynaptically connected with the hand area of primary motor cortex and paralleled by disynaptic (corticospinal) connections. The proportion of both depending on the primate species. Second, a large amount of cortico-cortical fibers directly connect the hand premotor cortex with several posterior parietal areas, which account for the precise sensorimotor control of hand movements, both by the somatosensory and the visual modalities.

With this in mind, the kinematic profiles of macaques’ grasp formation and reaching have been found to be strikingly similar to those in human prehension [9–19]. The prehension movements carried out by humans and these non-human primates are characterized by a single arm velocity peak occurring at about 50% of movement time together with a biphasic pattern of grip formation (i.e., the maximum finger aperture followed by a gradual closure of the grip) which is scaled to object size. It is therefore likely that the maximum grip aperture represents a widespread feature of object acquisition, which reflects the encoding, by the visual system, of the physical parameters of the object [1].

An aspect, however, that needs to be further clarified in macaques is how the two components of prehension, reaching and grasping, are coupled functionally. In particular, it remains unclear through which coordination mechanisms the two components of prehension do interact [11]. In humans, this issue has been tackled by experimentally imposing constraints on reaching [20, 21]. As an example, the maximum distance between the thumb and the index finger as the hand moves toward an object depends on the speed at which the movement is completed. When a subject tries to reach for an object quickly there is an increase in reaching error, which is compensated by naturally spreading his/her fingers farther apart than when he/she is trying to reach for the same object at a slower pace [21]. This has been taken as an evidence that, in advance of movement, formation of grasp is planned to take into account not only the perceived characteristics of the object but, also, internalized information based on past experience about the likely accuracy of the reaching component [21]. In other words, spatial links exist between the reaching and the grasping components in the sense that spatial aspects of grasp formation depend on spatial aspects of the reaching component.

In order to further characterize the kinematic organization of macaques’ prehension and how it compares with humans’, the study presented here was designed to systematically investigate reach-to-grasp movement kinematics in free-ranging macaque monkeys as they snatched food items from one another (i.e., snatching condition) or in the absence of threatening competitors (i.e., unconstrained condition). The snatching action offers an ideal opportunity to uncover what mechanisms are used by this species to synchronize and coordinate reaching and grasping components because they are performed at a higher speed with respect to unconstrained occasions. This condition, in terms of speed differences, might be comparable to human studies in which movements performed at fast and normal speed are compared [21].

We predict that if the kind of intersegmental coordination between the two components suggested for humans applies to macaques, then any possible increase in reaching error during snatching will be compensated at the level of the grasping component.

## Materials and Methods

### Ethics statement

The experimental procedures were approved by the Institutional Review Board for Animal Research at the University of Padua (Organismo Preposto al Benessere Animale; O.P.B.A.), and were in accordance with the recommendations of the Weatherall report, "The use of non-human primates in research”. We state that (i) no interaction or interference with the animals occurred; (ii) no animals were housed in captivity for this study; (iii) no animals were subjected to invasive procedures, or were sacrificed for this study; and (iv) no specific permission was required from the Moroccan authorities for this study.

### Species studied

Six juvenile macaque monkeys (*Macaca sylvanus;* common name: *Barbary Macaque*) with an estimated age of no more than four years. The age of individuals was estimated according to Fa [22]. All individuals belong to a fission and fusion troop of about 25 individuals part of a largest population of *Macaca sylvanus* studied by Camperio Ciani and colleagues [23, 24] since 1983. During the selected video sequences (2 hours) 18 individuals were visible. The others were possibly in the nearby forest foraging. The troop live in the Azrou cedar forest located in an area called La Carriere Toumliline, Morocco (Middle atlas—Mixed Cedar and Oak forest near the town of Azrou, 33.15° N; 5.15° W). Juveniles were studied since this age class is the one that most frequently performs snatching behavior with respect to adult individuals, suggesting that this peculiar behavior is typical of this age class of individuals. The troop composition (adults and juveniles) was determined on the basis of the shooting sequences examined.

### Data collection

A total of 12 hours of video footage was filmed using a digital camcorder (GoPro camera Hero3 Black Edition; sampling rate 100 Hz) during daylight hours (exclusively between 10.00 and 13.00) between the 2^nd^ and the 12^th^ of September 2013. The hidden camera was located at 10 m distance from the monkeys and commanded via a remote control. In view of the difficulty of filming any particular monkey grasping an object for any length of time before it moved away or turned its back, continuous sampling was considered the more appropriate method to film the individuals being observed [25]. As the study aimed to capture the animals in their natural habitat, a zoom lens was utilized in order to film them from a distance as they sat on the ground or moved from place to place going about their normal activities. Just as has been previously described in the literature [9–12; 16–19], the monkeys’ movements were filmed in the sagittal plane to avoid motion artifacts. It is important to underline that all the objects that were grasped were indigenous to that particular geographic area which was naturally scattered with green oak acorn and nuts. Our attention was focused on analyzing movements directed towards those objects/food items leading to precision grips.

### Grip classification

The precision grips appearing in the video sequences were classified depending on the areas of skin surface making contact with the objects being grasped. Two operators who were unaware of the study hypothesis and blinded to the experimental conditions were instructed to classify the grips as pinched (precision) when the distal pad of the thumb was opposed to the radial side of the index finger. Cohen’s Kappa value of 0.79 confirmed inter-rater reliability. It is nevertheless important to remember that in natural situations/environments, spontaneous movements do not necessarily fit into classical precision grip categories: at times three fingers may be involved, at others various finger combinations which often flow fluidly from one configuration to another can be observed. For the sake of comparison with previous studies [9–12; 16–19], only the precision grip movements, in which the monkey used exclusively the thumb and index fingers, were analyzed. The present study was exclusively concerned with right hand grasping movements. The laterality quotient (LQ) was 69 (± 15) with a LQ of 100 reflecting a full right-hand preference.

### Activity classification

**Fig 1:**
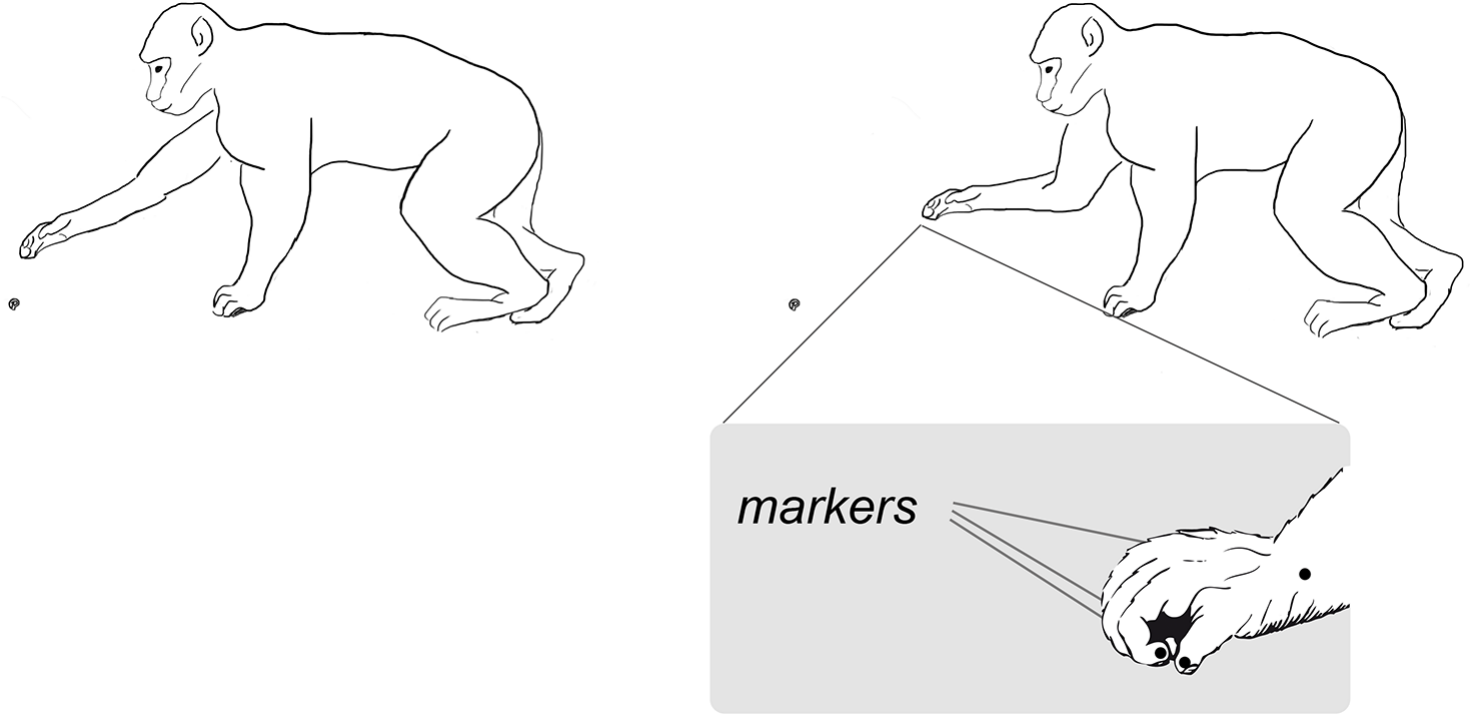
Graphical representation of the monkeys' postural conditions. Schematic drawing of the reach-to-grasp movement performed by the monkey (from right to left). Marker positions upon the hand are illustrated in the callout.

The movements that were analyzed and compared regarded two different situations: 1) unconstrained grasping or 2) snatching. For the former, the monkey was filmed as the animal interrupted an ongoing locomotor activity, briefly stopped in a tripedal position, and grasped an object of interest without any competition or interaction with other macaques (see Fig 1). The animal’s stability was determined by the locomotor pattern utilized. In this case, the specific pattern adopted by the monkey as it approached the item to be grasped was a diagonal sequence gait, that is when the hind foot touchdown is followed by that of the contralateral or opposite-side hand [26]. This information is important given that the feet and hand forming the tripod supports the animal’s body weight distribution and gives it stability when it comes to a stop (i.e., the monkey frozen in position). A similar context was analyzed for the snatching’ condition, i.e. an individual was filmed as it approached an object, stopped briefly in a tripedal position, and started the movement to snatch it. For both conditions the onset of the reach-to-grasp movement was defined as the time the individual’s arm/hand not forming the tripod of support rose from the ground and began the reach-to-grasp action. At the time the reach-to-grasp action began the object was approximately at 20 cm ± 0.3 cm away from the individual for both the unconstrained and snatching conditions.

### Data analysis

The video sample was analyzed frame-by-frame using a software developed to perform two-dimensional (2D) post-hoc kinematic analysis (AB-ACUS Technologies, Milan, Italy). Markers were inserted manually post-hoc via software on the anatomical landmarks of interest on the videos (Fig 1) and were tracked throughout the time course of the movement sequence. As shown in Fig 1, markers were positioned on the wrist of each individual so we could calculate the reaching component and on the nails of its index fingers and thumb so we could calculate the grip aperture (i.e., the distance in mm between the marker positioned on the index finger and the marker positioned on the thumb) as a function of time [9–11; 16–19]. The onset of movement for both the unconstrained and snatching conditions was defined as the time the tangential velocity of the wrist marker crossed a threshold (5 mm/s) and remained above it for at least two frames. The end of the movement was defined as the time the fingers closed around the object and there were no further changes in the distance between the index finger and the thumb. To avoid any skewing effect, only time frames in which reaching movements were performed along a plane that was perpendicular to the camera axis and in which the animal was located in the central part of the image were selected and analyzed. The positioning of the video camera axis and the plane of motion were verified by measuring the length of selected bone elements (e.g., forearm). This procedure was utilized to guarantee a constant point of reference during movements taking place on the plane perpendicular to the camera axis. A frame of reference identifying respectively *X* and *Y* axes as horizontal (ground) and vertical directions was manually set by the operator. A known length item within the camera’s field of view and in the same plane as the movement observed was used as the reference measurement unit. Movement tracking procedures were performed to extract a number of kinematic parameters. In accordance with previous studies focusing on macaques [9–11; 16–19], the following dependent measures were considered: (i) the movement time (i.e., the time between movement onset and when the monkey’s hand grasped the object); (ii) the wrist peak velocity (i.e., the time and amplitude of the reach peak velocity); (iii) the deceleration time (i.e., the time from wrist peak velocity to the end of the movement; (iv) the break-point (i.e., a plateau measured along the velocity profile determined for each trial) was determined by looking for two consecutive samples (at least) in which the velocity remained constant after it had started to decrease; (v) the maximum grip aperture (i.e., the time and the amplitude of the maximum distance between the thumb and index finger calculated from the beginning of reaching). A Linear-phase Autoregressive Model-Based Derivative Assessment algorithm (LAMBDA filter; [27, 28]; cutoff frequency, 10 Hz) was used for differentiating the marker position as to obtain velocities. Ten movements of each of the two conditions being considered were randomly chosen from a larger sample for each of the individuals being studied. The mean values of each dependent measure were calculated for each condition for each subject. The temporal values of the velocity and the grip profiles found in the two conditions were also normalized with respect to the movement duration. The accuracy of the measurement depends on the size of the field of view of the camera and the resolution of the sensor in terms of pixels. In the specific case, accuracy is 3 mm. In terms of maximum grip aperture this translates in an error of ± 0.4 mm. Mean values were entered into a separate analysis of variance (ANOVA) with the type of action (unconstrained, snatching) as within subject factors. To further test the variability of the reaching component for the two types of actions, the accuracy of the reaching was assessed at two specific time-points: at the time of the maximum grip aperture and three frames before contact was made (i.e., pre-contact). The perpendicular deviation of the wrist trajectory from the virtual line linking the starting position with the target was adopted as an accuracy index [21]. The standard deviation (SD) of that distance, that is the Variable Error, was computed over ten trials of each condition for each subject. A two-way repeated measure ANOVA on the SDs with the type of action (unconstrained; snatching) and the time of measurement (maximum grip aperture; pre-contact) was performed. All the main assumptions behind this statistical model (i.e., normality and sphericity) were checked before running the ANOVAs. The Kolmogorov-Smirnov test revealed that the normality assumption was satisfied (α-level: .05), and the Mauchly test showed that the sphericity assumption was not violated (α-level: .05). The post-hoc pairwise comparisons of the SD analysis were carried out using *t*-tests, and the Bonferroni adjustment for multiple comparisons was applied. To test the level of intersegmental coordination between the components, Pearson’s correlation coefficients were applied. The correlation between the onset of the low velocity phase of the arm trajectory (see the results section) and the time of maximum grip aperture was specifically tested.

## Results

### Reaching and grasping components

**Fig 2:**
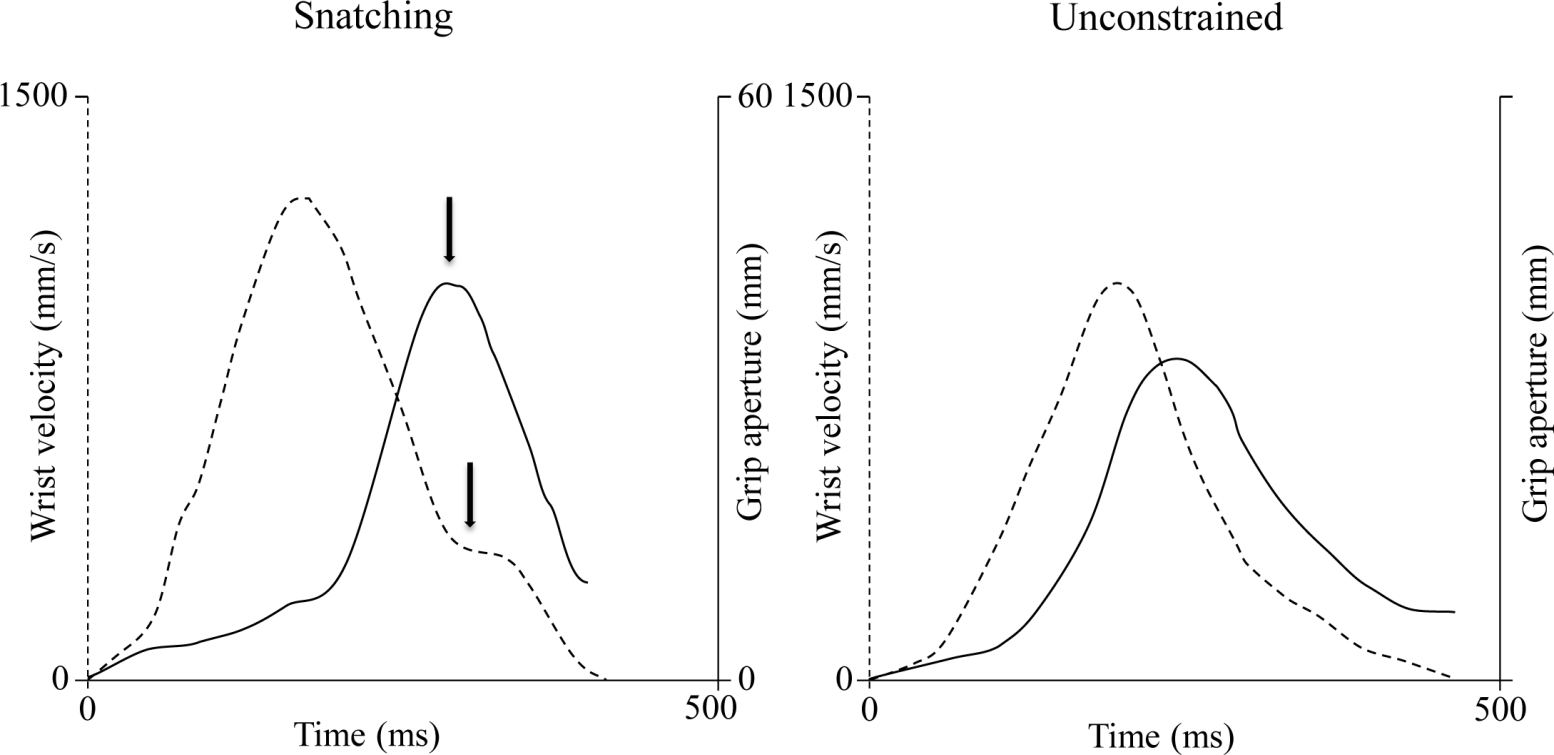
Superimposition of the velocity and grip profiles for the snatching and the unconstrained conditions. Arrows indicate the correspondence between the time at which the maximum grip aperture and the beginning of the low velocity phase occur for an individual movement for the snatching condition. Dashed lines = velocity profiles. Solid Lines = grip profiles.

The type of action (snatching vs. unconstrained) affected both the reaching and the grasping components. In terms of movement time, snatching movements were shorter than unconstrained movements [357 ± 38 vs 438± 41 ms, respectively; *F*(1,5) = 8.22, *P* = 0.001, *η*_*p*_^*2*^ = 0.70]. In agreement with previous literature [9–11; 16–19], wrist velocity profiles were approximately bell-shaped (Fig 2). The wrist peak velocity was significantly higher for the snatching than for the unconstrained condition [956 ± 98 mm/s vs 776 ± 81 mm/s; *F*(1,5) = 37.22, *P* < 0.0001, *η*_*p*_^*2*^ = 0.78].

Moreover the wrist peak velocity was reached earlier for the snatching than for the unconstrained condition (absolute terms: 161 ± 21 ms vs 215 ± 20 ms; *F*(1,5) = 54.21, *P* < 0.0001, *η*_*p*_^*2*^ = 0.80; relative terms: 45 ± 4% vs 49 ± 5%; *F*(1,5) = 31.06, *P* < 0.0001, *η*_*p*_^*2*^ = 0.84), thus suggesting a longer deceleration phase (from peak velocity to the end of the movement).

**Fig 3:**
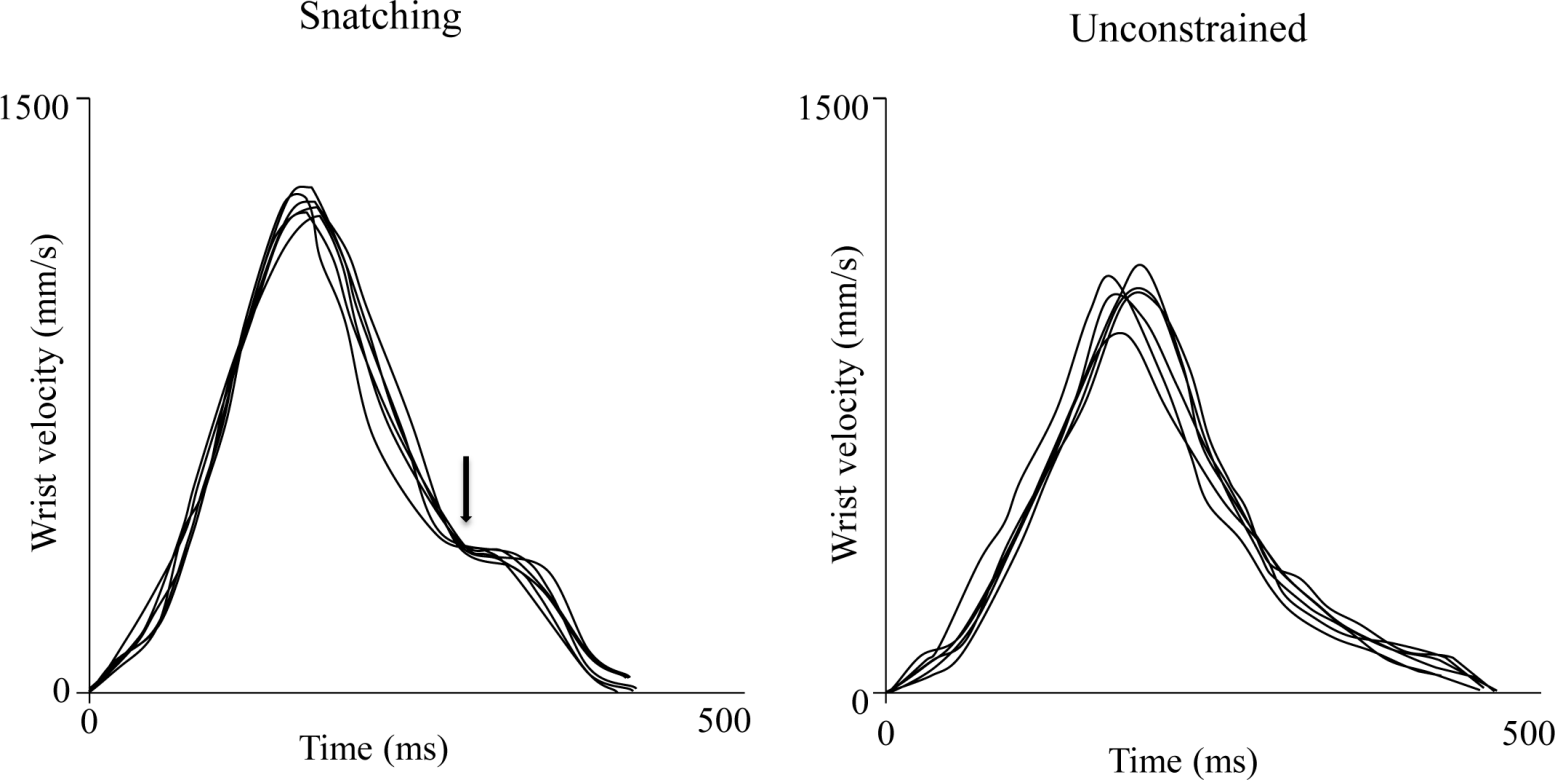
Superimposition of the velocity profiles for the snatching and the unconstrained conditions for the six individuals considered. This figure well represents how stereotypical this type of movements was with specific reference to the break point occurring along the deceleration phase. The arrow indicates the beginning of the break point.

Along the deceleration phase, a break-point occurring on the average at 80% of the movement duration was observed only for the snatching condition (Fig 2). This break-point was a systematic event occurring in 98% of the total number of the snatching movements considered (Fig 3). As concerns the grasping component, two phases were distinguished: a finger extension (grip formation), followed by a finger flexion (actual grasping). As shown in Fig 2, the maximum grip aperture was significantly greater [32 ± 3 mm vs 23 ± 2 mm; *F*(1, 5) = 29.32, *P* < 0.0001; *η*_*p*_^*2*^ = 0.76], and it was reached significantly later in both absolute [289 ± 32 ms vs 315 ± 26 ms; *F*(1, 5) = 17.42, *P* < 0.0001; *η*_*p*_^*2*^ = 0.81] and relative [81 ± 7% vs 72 ± 5%; *F*(1, 5) = 23.18, *P* < 0.0001; *η*_*p*_^*2*^ = 0.80] terms for the snatching than for the unconstrained grasping condition.

### Accuracy of the transport component

The averaged SDs of wrist trajectory deviation (variable error) for each subject are listed in Table 1. The ANOVA on the variable error revealed a statistically significant interaction between the type of action and the time of measurement [*F*(1,5) = 11.37, *P* < 0.001; *η*_*p*_^*2*^ = 0.72]. For the snatching condition, post-hoc contrasts indicated a significant increase of variance at the time of maximum grip aperture than at pre-contact (*P* < 0.05; see Table 1). For the unconstrained grasping condition, no differences in variance at the time points considered were detected (*P* > 0.05; see Table 1). At the time of maximum grip aperture, the wrist trajectory variable error was greater for the snatching than for the unconstrained grasping condition (*P* < 0.05; see Table 1). At pre-contact time no differences were found between the snatching and unconstrained grasping conditions (*P* > 0.05; see Table 1).

**Table 1:** Average SD (Variable Error) in mm in wrist Y-Position at Maximum Aperture and Pre-Contact for each subject. The corresponding mean grip aperture in millimeters is reported in parentheses.

Position in trajectory	Maximum Aperture		Pre-contact	
Type of Movement	Unconstrained	Snatch	Unconstrained	Snatch
Subject				
1	3.1 (24 ± 2)	4.9 (33 ± 4)	3.4 (19 ± 2)	3.2 (28 ± 3)
2	2.9 (23 ± 2)	4.1 (35 ± 3)	3.3 (16 ± 3)	3.1 (30 ± 4)
3	3.1 (21 ± 3)	3.8 (32 ± 4)	3.1 (16 ± 2)	3.0 (28 ± 3)
4	2.9 (25 ± 4)	4.7 (33 ± 5)	3.7 (18 ± 3)	3.5 (27 ± 5)
5	2.6 (24 ± 3)	4.9 (31± 3)	3.2 (20 ± 4)	3.6 (26 ± 3)
6	2.8 (24 ± 2)	4.4 (31± 2)	2.9 (16 ± 3)	3.2 (24 ± 4)

### Correlation analysis

The time of maximum grip aperture for the snatching condition was synchronized with the beginning of the break-point detected along the velocity profile (Fig 2). The correlations calculated over all the snatching trials and separately for each subject are shown in Table 2. A significant correlation was found for all the subjects. There appears thus to be a high degree of temporal coupling between the reaching and the grasping components for the snatching condition.

**Table 2:** Significant values for the considered correlation for each subject.

Subjects	Time of maximum grip aperture/Start of the break-point
1	*r* = 0.81, p < 0.01
2	*r* = 0.90, p < 0.01
3	*r* = 0.88, p < 0.01
4	*r* = 0.82, p < 0.01
5	*r* = 0.89, p < 0.01
6	*r* = 0.71, p < 0.01

## Discussion

In reaching for an object in the environment, spatiotemporal links between hand configuration (grasping) and arm movement (reaching) are required to allow for a correct unfolding of the hand before it arrives at the target object. It has been argued that in humans there is a tightly defined relationship between certain points on the grasp and reach trajectories [21]. This study focused on examining if such spatiotemporal relationship between components also characterizes prehensile actions of macaque monkeys. The analysis of our results showed that during snatching conditions, wrist variability is associated with maximum finger aperture, and they are both greater than those found for the unconstrained condition. Just as in humans performing quick reaching movements, opening the hand wider may provide increased tolerance for positioning errors due to the higher wrist variability [21]. The increase in hand aperture might be interpreted as an error-compensating adjustment, to avoid that thumb or fingertip collides with the object.

But how is this synchronization achieved? Notably, wrist variability for snatching movements was higher during maximum grip aperture, but similar to that noticed for the unconstrained condition when measured just before contact. Therefore the increase in maximum grasp aperture during snatching might be considered predictive, centrally patterned and not based on concurrent feedback. Further support to this inference comes from the evidence that during snatching the time of maximal hand aperture correlates with a break-point along the deceleration phase of the wrist velocity profile which occurs at about 80% of the reaching time. This precise synchronization of reaching and grasping parameters is an unexpected and relevant finding. It gives us an idea on how in macaques the proximal and distal movements could be also temporally coordinated. Such break-point is remindful of an observation made by Jeannerod [2] in a study looking at the kinematical pattern of natural prehension in humans. He reported that the deceleration of the arm trajectory was consistently marked by a break-point where the tangential velocity tended to become constant before the movement was stopped at the contact with the object. This break-point occurred within 70–80% of completion of movement and it was correlated with the time at which participants reached the peak of maximum grip aperture. This event has been interpreted as a positioning or target acquisition phase, a necessary constraint for movements involving several segments and requiring a *high degree of precision*. In the present results, the correlation between the time at which the break-point along the deceleration phase of the velocity profile starts and the time of maximum grip aperture might indicate the existence of a control system that interweaves activations between separate motor schema as to coordinate movements involving several segments and requiring a high degree of accuracy (such as grasping small objects quickly). Notably, the above mentioned correlation occurs at the time the wrist variability is at its highest and it is associated with a finger aperture which is greater than that found for the unconstrained condition. Overall, these findings suggest that a higher order program governs both the spatial and temporal coordination of the two components of prehensile actions during highly demanding conditions, such as snatching small targets.

In neural terms, neurophysiological and lesion data suggest that in both humans and macaques, reaching and grasping are mediated by two separate anatomical pathways (for review see [29]). Grasping is organized by a lateral parieto-frontal circuit involving the anterior intra-parietal area and ventral premotor area, and reaching by a more medial parieto-frontal circuit including medial intraparietal area and dorsal premotor area [29]. Recent evidence, however, shows that the medial parieto-frontal reaching circuit in addition to playing an important role in organizing reaching towards the location of an object is also involved in grip formation [30, 31]. This suggests that this specific area is involved in the control of both reaching and grasping circuits. In this connection, the present findings might indicate that when reaching is unstable during the execution of a fast movement, a break point along the deceleration phase might reflect an additional activity recruitment necessary to synchronize and coordinate the two components by this neural system.

## Conclusions

The data presented here are based on precise measurements of spontaneous grasping movements by animals acting in their natural environment. Our data show for the first time that the control mechanisms underlying reaching and grip formation in macaques are affected by task constraints such as movement speed and grip accuracy. These results are consistent with findings in humans suggesting that task constraints can modulate the proximal and distal components of a coordinated action. Based on these findings, it can be hypothesized that when monkeys perform reaching movements faster than normal the coordination between the reaching and grip components of prehension involves both spatial and temporal coupling. A higher order control system seems to be responsible for solving coordination problems due to variations in the spatial positioning of the arm. When reaching for a small target is carried out quickly, the failure to reduce variability as the target is being approached calls for coordination strategies amongst components, which might serve to partially dissipate errors.
